# *Serratia marcescens* outbreak in a neonatal intensive care unit: crucial role of implementing hand hygiene among external consultants

**DOI:** 10.1186/s12879-014-0734-6

**Published:** 2015-01-13

**Authors:** Carlotta Montagnani, Priscilla Cocchi, Laura Lega, Silvia Campana, Klaus Peter Biermann, Cesare Braggion, Patrizia Pecile, Elena Chiappini, Maurizio de Martino, Luisa Galli

**Affiliations:** Department of Health Sciences, University of Florence, Viale Pieraccini 23, 50139 Florence, Italy; Anna Meyer Children’s University Hospital, Viale Pieraccini 23, 50139 Florence, Italy; Microbiology and Virology Unit, Careggi University Hospital, Florence, Italy

**Keywords:** *Serratia marcescens*, Outbreak, Neonatal intensive care unit, Molecular epidemiology, Ocular colonization, Rectal colonization, Hand hygiene

## Abstract

**Background:**

*Serratia marcescens* represents an important pathogen involved in hospital acquired infections. Outbreaks are frequently reported and are difficult to eradicate. The aim of this study is to describe an outbreak of *Serratia marcescens* occurred from May to November 2012 in a neonatal intensive care unit, to discuss the control measures adopted, addressing the role of molecular biology in routine investigations during the outbreak.

**Methods:**

After an outbreak of *Serratia marcescens* involving 14 neonates, all admitted patients were screened for rectal and ocular carriage every two weeks. Extensive environmental sampling procedure and hand sampling of the staff were performed. Antimicrobial susceptibility pattern and molecular analysis of isolates were carried out. Effective hand hygiene measures involving all the external consultants has been implemented. Colonized and infected babies were cohorted. Dedicated staff was established to care for the colonized or infected babies.

**Results:**

During the surveillance, 65 newborns were sampled obtaining 297 ocular and rectal swabs in five times. Thirty-four *Serratia marcescens* isolates were collected: 11 out of 34 strains were isolated from eyes, being the remaining 23 isolated from rectal swabs. Two patients presented symptomatic conjunctivitis. Environmental and hand sampling resulted negative. During the fifth sampling procedure no colonized or infected patients have been identified. Two different clones have been identified.

**Conclusions:**

Ocular and rectal colonization played an important role in spread of infections. Implementation of infection control measures, involving also external specialists, allowed to control a serious *Serratia marcescens* outbreak in a neonatal intensive care unit.

## Background

*Serratia marcescens* (*S. marcescens*) was described for the first time in 1819 and thought to be a non-pathogen until the latter half of the 20^th^ century [1]. Nowadays this microorganism is an accepted clinical pathogen, causing pneumonia, urinary tract infections, septicemia and meningitis, particularly in high risk settings [2]. Antimicrobial resistance represents a real challenge for hospital settings, considering that many microorganisms including *S. marcescens* carry both chromosomal and plasmid-mediated resistance determinants, such as extended spectrum beta-lactamases (ESBLs). Acquired resistance of *S. marcescens* to several antimicrobial agents, including narrow-spectrum beta-lactams and colistin, was well-described [1]. Resistance determinants can easily spread from a species to another, a further reason to implement infection control measures in hospital settings [3,4]. *S. marcescens* is an ubiquitous pathogen, whose complete eradication from the environment is often difficult: a *plethora* of sources of infection are reported, including healthcare workers hands, infected neonates and contaminated medical devices [1]. In hospitalized adults *S. marcescens* tends to colonize respiratory and urinary tracts, whilst in neonatal intensive care units (NICUs) gastrointestinal tract represent an important reservoir for cross-contamination [5]. Hospitalized newborns are particularly susceptible to bacterial conjunctivitis, and infections on the eye are an area where *S. marcescens* stands out as a pathogen [1]. Several outbreak reports concluded that colonized and infected infants represent the most important reservoir of *S. marcescens* and, once affected, many neonates appear to remain colonized for long periods despite antibiotic treatment [6]. The early detection of colonized or infected patients and the prompt implementation of infection control measures are significant factors in the control of bacterial spread. To obtain this goal, an accurate description of outbreaks appears to be crucial. Thus, the aim of this study is to describe an outbreak of *S. marcescens* in a NICU and discuss the control measures adopted, addressing the role that molecular biology could play in routine investigations of outbreaks.

## Methods

### Surveillance and environmental investigations

During a *S. marcescens* outbreak in a NICU, all admitted babies were screened for gastrointestinal and ocular carriage every two weeks. Moreover, extensive environmental sampling and screening of the staff were performed. Cultures where obtained from other sites, as appropriate, in symptomatic patients. Ethical approval was not required because all swabs were carried out for managing the outbreak, according criteria of good clinical practice.

### Bacterial culturing and identification

Samples were inoculated onto bacteriological culture media, then incubated at 37°C for 24 hours. *S. marcescens* strains were identified using Vitek2 (bioMérieux, Marcy L’Etoile, France) GN cards.

### Antimicrobial susceptibility testing

The isolates were tested by Vitek (Vitek 2, bioMérieux, Marcy L’Etoile, France), using N202 card and antimicrobial susceptibility was interpreted according to the EUCAST breakpoints.

### Molecular analysis

Chromosomal DNA was extracted using QIAamp DNA Mini Kit (QIAGEN N.V., Netherlands). Intra-specific relatedness was investigated by means of Enterobacterial Repetitive Intergenic Consensus PCR (ERIC-PCR) fingerprinting as previously described [7]. Resistance genetic determinants were tested using different methods. We used the rapid and accurate PCR assay by Colom and colleagues to test the presence of blaTEM, blaSHV and blaOXA genes [8]. CTX-M ESBL presence was investigated using protocol published by Woodford and colleagues [9].

### Definition of infection and colonization

We defined “infected” those patients who showed clinical signs of conjunctivitis and positive ocular swabs, and “colonized” patients testing positive for rectal or ocular swabs without clinical signs.

## Results

### Description of the outbreak

An outbreak of *S. marcescens* was identified from May to November 2012 in a 22 cots tertiary NICU. The ward has a volume of approximately 9000 annual patient days and is located in a six years old building. Patients admitted in the ward are mainly complex surgical cases. Before starting surveillance, *S. marcescens* was isolated from blood in four patients, from intestinal stoma in two patients and from ocular secretion in further six newborns. An infection control meeting was planned between the NICU staff and the infection control committee to establish infection control measures. The index case was identified in a newborn with sepsis, transferred from another hospital. NICU staff was pushed into implementing effective hand hygiene with 70% ethyl alcohol hand sanitizer or 4% chlorhexidine gluconate solution and gloves use. Extensive environmental sampling, including sinks, soap and chlorhexidine dispensers, sluices, cots, ventilators, balances, stethoscopes, clinical charts, ophthalmoscopes, computer keyboards and mouses and hand plates of staff were all negative for *S. marcescens*. However, samples from medical charts, computer keyboards and mouses revealed the presence of several microorganisms, including *Staphylococcus* spp., *Enterococcus* spp., *Pantoea* spp. and *Sphingomonas paucimobilis.*

Sixty-five NICU patients were sampled obtaining 297 ocular and rectal swabs in five times: at least two ocular and one rectal swabs were obtained from each patient. Eighteen out of 65 patients tested positive for *S. marcescens*, being seven of them positive more than once. During the first two procedures, 120 samples were analyzed, from 26 patients. The first sampling procedure revealed one rectal positive patient, who has previously experienced *S. marcescens* conjunctivitis. The other patients infected before starting surveillance have been already discharged at time of procedure samplings. During the second procedures, other two patients resulted rectally colonized and one patients both ocular and rectally colonized. The third procedure revealed 12 out of 20 patients tested positive for *S. marcescens* (ten of whom previously sampled): five rectal, two ocular and five both rectal and ocular. Two out of the ocular colonized patients were symptomatic. Following the worsened result of these sampling procedure and lack of isolation of *S. marcescens* from environmental samples, new implementation of effective hand hygiene was performed, involving all the external specialists. Therefore, all the consultants, especially surgeon, cardiologists and ophthalmologists, were invited to strictly observe infection control measures, including hand hygiene, gloves use and accurate disinfection of personal medical devices. Colonized and infected babies were cohorted. Dedicated staff was established to care for the colonized or infected babies. Daily control of the implemented measures was performed. Ocular colonized or infected newborns were treated with topical ofloxacin.

During the fourth sampling procedure, 19 patients were tested. Three patients tested positive for both ocular and rectal swabs and five only rectal.

A fifth patient screening did not reveal any positive samples. Tables 1 and 2 resume sampling procedure results.

**Table 1:** **Results of sampling procedures**

**Sampling procedure**	**Number of patients**	**Number of positive patients**	**Number of samples**	**Only ocular positive**	**Only rectal positive**	**Both positive**	**Clone A**	**Clone B**
	**(not previously tested)**	**(not previously resulted positive)**		**(not previously isolated)**	**(not previously isolated)**	**(not previously isolated)**		
1	21 (20)	1	63	-	(1)	-	1	-
2	19 (5)	4 (3)	57	-	3 (2)	(1)	5	-
3	20 (10)	12 (11)	60	(2)	5 (4)	(5)	16	1
4	19 (11)	8 (3)	57	-	5 (1)	3 (2)	4	7
5	20 (18)	-	60	-	-	-	-	-
Total	99 (65)	25 (18)	297	2	14 (8)	9 (8)	26	8

**Table 2:** **Results of sampling procedures according to patients and date of screening**

**Patient**	**3/09/2012**	**17/09/2012**	**3/10/2012**	**22/10/2012**	**6/11/2012**
A	-	N	RP	-	-
B	-	N	N	RP	-
C	-	RP	-	-	-
D	-	N	RP	-	-
E	-	-	OP	-	-
F	RP	RP	-	-	-
G	N	N	ORP	N	N
H	-	-	-	ORP	-
I	-	N	RP	-	-
L	N	ORP	-	-	-
M	N	RP	RP	-	-
N	-	N	RP	RP	-
O	-	N	ORP	-	-
P	-	-	-	ORP	-
Q	N	N	ORP	RP	-
R	-	-	ORP	ORP	-
S	N	N	OP	RP	-
T	N	N	ORP	RP	-

N: negative; RP: rectal positive; OP: ocular positive; ORP: ocular and rectal positive; −: not admitted.

Following microbiological investigation and implementation of infection control measures, this outbreak was brought under control and no further infected or colonized patients were identified. No serious infections were reported during the investigational period. All infected patients recovered without *sequelae*.

### Microbiological results

Thirty-four *S. marcescens* isolates were collected during the whole sampling procedures, from 25 patients: 11 out of 34 strains were isolated from eyes, being the remaining 23 rectal swabs.

Antimicrobial resistance patterns were substantially maintained during the observational period; all isolates were resistant to colistin and amoxicillin-clavulanic acid and all strains but two showed resistance to gentamicin. Four strains showed resistance against ceftazidime and two against piperacillin-tazobactam. All isolates were sensitive to other beta-lactam antibiotics. ERIC-PCR fingerprinting results highlighted the presence of two different clones. One of these two clones, named A, was more represented than the other clone named B, infecting 14 out of eighteen patients. Strains sampled from different body sites of the same patient belonged to the same clone in all but two cases. Clone A was also responsible for one of the sepsis case occurred before starting surveillance. We were not able to perform ERIC-PCR fingerprinting of *S. marcescens* strains involved in the other infections occurred before starting surveillance. The clones did not differ significantly in terms of antimicrobial susceptibility: resistance pattern of clone A and clone B were similar, being difficult to distinguish among these two clones basing on antimicrobial susceptibility only. Molecular tests for resistance genetic determinants were negative.

## Discussion

*S. marcescens* has been reported to be responsible of 15% of nosocomial infection in NICU [10]. Most of the published studies concluded that colonized and infected infants are the most important *reservoir* of *S. marcescens* [2]. Once affected, most of the infants appear to remain colonized, especially in their gut, for long periods [2].

This report describes a successfully controlled serious *S. marcescens* outbreak in a tertiary referral NICU. The index case was a newborn transferred from another hospital, presented with *S. marcescens* sepsis. Despite extensive environmental and staff sampling, we were not able to determine an environmental *reservoir* of infection. The asymptomatic colonized neonates were the most important *reservoir* of bacteria in this outbreak and transient contaminated hands probably the most important vehicle. Overall, 14 babies resulted infected (12 before starting surveillance and two thereafter) and other 16 ocular or rectally colonized.

Similarly to what previously described [5,11], in our outbreak gastrointestinal colonization was the most important reservoir of *S. marcescens*, counting 17 colonized patients.

Conjunctivitis is one of the most common healthcare-associated infection in newborns, due to anatomical reasons, immaturity of immune systems and eye colonization [12]. A recent epidemiological study of causes of conjunctivitis in a NICU conducted in Portugal, revealed *S. marcescens* as the most common pathogens, being responsible of 27,9% of cases [13]. Since the high rate of conjunctivitis identified during the outbreak, we decided to perform eye surveillance cultures from all admitted neonates. Ten newborns resulted positive, among which only two were symptomatic. Interestingly, one screened patient tested negative for colonization in the morning, whereas developed clinical signs of conjunctivitis in the evening. The sample obtained by ocular secretion resulted positive for *S. marcescens*.

*S. marcescens* typically survives well in soil and moist environments [1]. In several NICU *S. marcescens* outbreak, an environmental source of spread was found, including colonized hand wash soap or breast pumps [11,14,15]. An outbreak of *S. marcescens* conjunctivitis in a NICU was reported to be caused by infected bottle of ticlosan [16]. We performed an extensive environmental sampling procedure, which was negative for *S. marcescens*. Since the high rate of ocular colonized patient, we presumed a contamination of ophthalmic medical devices. Environmental samples from ophthalmoscopes and other ophthalmic devices were also obtained, but resulted negative. On the other hand, samples from medical charts, computer keyboards and mouses revealed the presence of several microorganisms. The high diffusion of contaminants on these surfaces suggests non-compliance with hand hygiene by healthcare workers.

NICU staff hand screening resulted negative, but we were not able to perform hand sampling from external consultants. However, we postulated that the most probably source of transmission was transient hand carriage after contact with colonized patients.

Use of alcohol-based hand sanitizer and chlorhexidine solution was implemented, as documented by increased consumption of these products. However, we did not obtain outbreak control after implementing hygiene procedures involving only NICU staff. Since mainly complex surgical patients are admitted in our NICU, several external consultants attend the ward, including surgeon, neurosurgeon, cardiologists and ophthalmologists. Therefore, we presumed that external consultants were less compliant with infection control measures and they were also pushed into implementing hygiene measures. After few weeks the outbreak was completely controlled. Hence, we concluded that external consultants had a crucial role in diffusion of *S. marcescens*.

Closure of the NICU was initially discussed, but it was ruled out for logistical reasons. Therefore, infected and colonized neonates were cohorted and cared by dedicated staff. No new serious cases have been documented since surveillance efforts have been implemented.

Differently from previous reports [5,11,17], all infected newborns recovered without *sequelae* and no serious infections have been reported during the surveillance period, in spite of a high rate of colonized patients.

It was possible to identify two genetically different strains of *S. marcescens* characterized by means of ERIC-PCR, a reliable tool for outbreaks investigations. The presence of two different clones was already demonstrated in a paper describing that an invasive *S. marcescens* clone was replaced by a less pathogenic strain [5]. In this case, we describe the simultaneous presence of two clones, being clone A responsible for the singular sepsis case we could report, for most of cases identified during the surveillance and for the two symptomatic conjunctivitis. Instead, clone B was isolated in four patients starting from the third surveillance procedure and was not involved in symptomatic cases. Therefore, we postulated that clone B was a less virulent strain, ubiquitous in the NICU, starting replace the clone A.

Despite the application of two different useful methods to detect antimicrobial resistance genetic determinants against beta-lactams, we were not able to find positive strains. Furthermore, more accurate molecular investigations concerning genetic determinants of resistance other than beta-lactam antimicrobial agents are needed.

## Conclusions

*S. marcescens* outbreak represents a serious challenge in hospital settings, above all in NICU. Frequently, *reservoirs* of infection were not identified. However, extensive surveillance procedures are essential in the infection control and molecular biology could help in understand spread of microorganisms. Ocular colonization, in addition to gastrointestinal colonization, could play an important role in outbreak maintenance. Implementation of standard measures, such as hand hygiene involving also external specialist are essential and often sufficient in obtaining control of the outbreak.
